# Diagnosis of acute pulmonary embolism by point-of-care ultrasound under continuous cardiopulmonary resuscitation: case series

**DOI:** 10.1016/j.ijscr.2025.111434

**Published:** 2025-05-13

**Authors:** Qishuo Zhang, Xiaomin Ou, Wanshan Liu, Lifeng Xiao

**Affiliations:** a Department of Medicine, Medical College of Wisconsin, Milwaukee, WI, USA; b Emergency Department, Shantou Central Hospital, Shantou, Guangdong, China; c Emergency Department, Cancer Hospital of Shantou University Medical College, Shantou, Guangdong, China

**Keywords:** Acute pulmonary thromboembolism, CPR, Point-of-care ultrasound, rTPA

## Abstract

**Introduction and importance:**

Acute pulmonary embolism, as one of the most common and dangerous diseases, sometimes can develop into cardiac arrest before receiving Computed Tomography Angiography (CTA) or other confirmatory workup. The success rate with merely Cardiopulmonary resuscitation (CPR) is extremely low, which raises the question whether it is appropriate to make medical decisions and treat patients empirically with systemic thrombolytic therapy based on point-of-care ultrasound.

**Case presentation:**

This case series reported three cases in which patients presented with cardiac arrest in the emergency department, given the cases were too emergent for CTA, pulmonary embolism was diagnosed based on point-of-care ultrasound given the emergency of the condition, the patient was given systemic thrombolytic therapy with continuous CPR, and achieved Return of Spontaneous Circulation (ROSC).

**Clinical discussion:**

These cases discuss the importance of bedside ultrasound in the rapid identification of acute pulmonary embolism and the importance of intravenous thrombolysis in the context of continuous cardiopulmonary resuscitation.

**Conclusion:**

Point of care ultrasound plays a crucial role in rapidly identifying acute pulmonary embolism in the emergency department. Systematic therapy with continuous CPR is an effective management for patients with cardiac arrest secondary to acute pulmonary embolism.

## Highlights

• This case series presents three patients who experienced cardiac arrest secondary to pulmonary embolism.
• Point-of-care ultrasound (POCUS) is the most direct and effective way to detect acute pulmonary embolism during ongoing cardiopulmonary resuscitation (CPR).
• Empiric intravenous thrombolytic therapy with Alteplase (rtPA) during continuous cardiopulmonary resuscitation is essential for achieving return of spontaneous circulation (ROSC) in case of suspected pulmonary embolism.

## 1 Introduction

Acute pulmonary embolism (APE) is one of the common causes of cardiac arrest, according to one study, there are 28 to 54 people in every 100, 000 people died of cardiac arrest. Among them, pulmonary thromboembolism accounts for 10 %. The incidence of cardiac arrest is 65–88 % among patients with massive pulmonary embolism [1,2]. Acute pulmonary embolism has various manifestations, which lack of specificity. Computed Tomography Angiography (CTA) is the golden test for diagnosis, however, some pulmonary thromboembolisms develop abruptly and may cause hemodynamic instability in the early stage [3], cardiac arrest, which requires cardiopulmonary resuscitation, during which CTA is infeasible. As a result, many cases are not diagnosed till autopsy [4]. Therefore, identifying PTE as soon as possible and providing appropriate treatment is the key to resuscitation. This report presented three cases of cardiac arrest on arrival of the emergency department, PTE was suspected given the finding of point-of-care ultrasound, patient received continuous CPR and also rTPA intravenously, ROSC was achieved, among them, one patient was cured and discharged, one of them were transited to comfort care afterward, the other was pronounced death after second cardiac arrest in the admission.

## 2 Method

This work has been reported in with PROCESS criteria [5].

## 3 Case report

### 3.1 Case 1

**Fig. 1:**
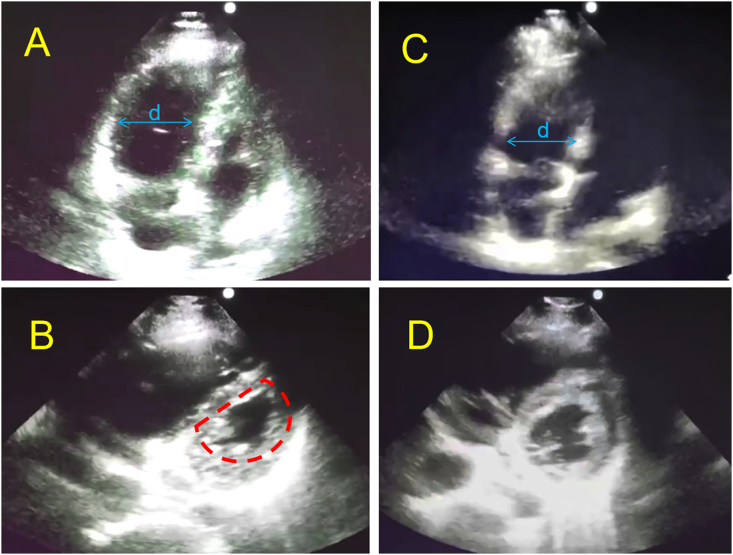
The cardiac point-of-care ultrasound of patient 1 before and after thrombolytic therapy. A: before thrombolytic therapy, apical five-chamber view: right ventricular significantly enlarged, RV: LV>1; B: before thrombolytic therapy, parasternal short axis， classic D shape sign, more obvious during systolic phase； C: after thrombolytic therapy (T + 0:70), RV size became smaller, RV: LV < 1, D: after thrombolytic therapy, IV septum deviation improved; D: shape sign disappeared.

**Fig. 2:**
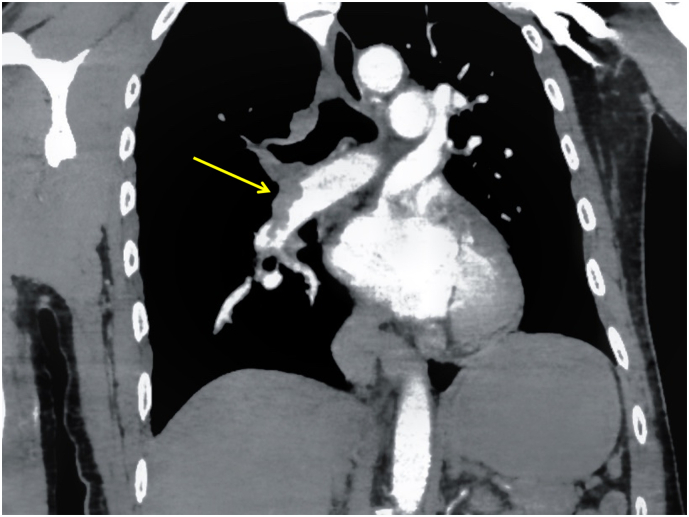
After thrombolytic therapy, CTA finding (T+ 1:36) there is still filling defects clots in the right upper lobe artery and multiple branches of the pulmonary artery.

A 49- year-old gentleman without significant past medical history presented to the emergency department with pleuritic chest pain, dyspnea, and cyanosis for 30 min. He recently got left side traumatic tibial plateau fracture was given external splint fixation and stayed at home for 5 days. On arrival, the patient was obtunded and in shock with BP 68/35 mmHg and hypoxia with SaO2 40 %, the patient was intubated and given intravenous fluid. Stat EKG and labs are shown below. EKG shows no ST elevation, troponin and BNP were both negative, D- dimer was elevated significantly to 2.53 μg/ml. point-of-care ultrasounds showed right ventricular significant enlargement, the parasternal short axis showed the classic D shape sign (Fig. 1 A, B, Video 1), which was more obvious in the systolic phase, and there is no visible thrombosis in the heart or lower limbs., however, given the history of recent trauma, there was still a high suspicion of acute pulmonary thromboembolism, CTA was planned but the patient developed progressive bradycardia then cardiac arrest (T 0:00). Telemetry shows a non-shockable rhythm. So the patient was started on CPR and systemic thrombolytic therapy empirically. The patient was given 10 mg rTPA intravenous bolus within 2 min (T+ 0:06), and then completed 90 mg via intravenous infusion within 120 min. Patient achieved ROSC after 3 doses of 1 mg epinephrine (T+ 0:19). His symptoms, BP and SaO2 were improved, point-of-care ultrasound showed decreased of right ventricular size and relief of the interventricular septum deviation (Fig. 1 C, D) (T+ 0:70). Afterward, patient was transferred to CCU and CTA (Fig. 2) was done, which showed clots formation on right upper lobular artery, left distal pulmonary artery trunk, left lower lobe main pulmonary artery, and distal branches involvement, as well as thrombosis in the right middle lobe pulmonary artery, right lower lobe pulmonary artery, and distal branches. On admission day 2, the patient was found to have coffee ground emesis from the nasogastric tube, Hb drops to 7.8 mg/Dl, diagnosis of acute upper gastrointestinal hemorrhage was made, the patient was given pure RBC transfusion for acute bleeding, emergent endogastroduodenoscopy (EGD) was done, which showed acute gastric and duodenal mucosal erosion and diffuse bleeding, homeostasis could not be achieved under EGD, patient was conservative treatment. His condition continued to progress, for which he developed cardiac arrest in the afternoon. The patient was unfortunately pronounced dead despite CPR and medical therapy.

### 3.2 Case 2

**Fig. 3:**
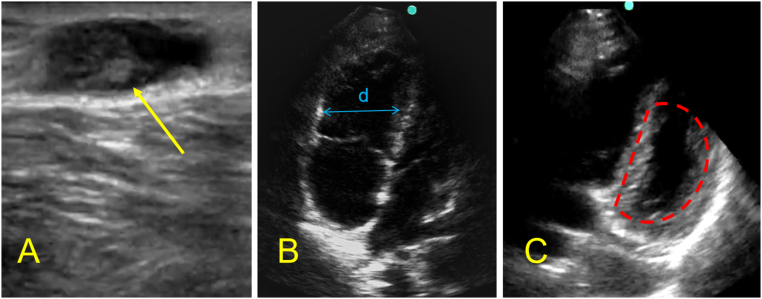
The lower extremity and cardiac point-of-care ultrasound of patient 2 after thrombolytic therapy. A: right great saphenous vein visible thrombosis (yellow arrow); B: apical four-chamber window shows significant enlargement of right ventricular (blue double arrow), RV: LV > 1, C: Parasternal short axis shows D shape sign (red dash line).

**Fig. 4:**
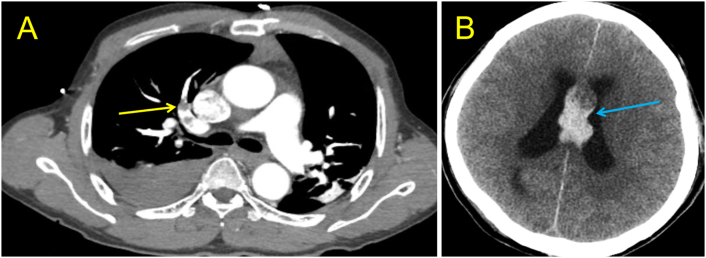
The CTA chest and CT head of patient 2 after thrombolytic therapy. A: after thrombolytic therapy, CTA shows right pulmonary artery filling defect (yellow arrow); B: On day 3 after thrombolytic therapy, CT showed septum pellucidum hemorrhage and broke into bilateral ventricles (blue arrow), brain edema with herniation formation.

A 65-year-old gentleman with a past medical history of atrial fibrillation and hypertension, not compliant with medications, presented to the emergency department with progressive chest tightness for half day, developed loss of consciousness 5 min before arrival, on arrival, the patient was found to have no spontaneous breathing and pulse (T+ 0:00), the patient was given CPR, intubation and mechanical ventilation, point-of-care ultrasound shows a significant enlargement of the right ventricle (Video 2), great saphenous veins visible clot (Fig. 3A), given the high suspension for acute pulmonary thromboembolism, patient was given systemic thrombolytic therapy (T+ 0:13), patient achieved ROSC after 5 doses of 1 mg epinephrine,. EKG and labs were done (Table 1) and a repeat point-of-care ultrasound was done, with significant right ventricle enlargement and D-shape size on the parasternal short axis (Fig. 3 B, C). CTA was done after the patient was hemodynamically stable (T+ 1: 38), which shows a right pulmonary artery filling defect (Fig. 4D). He was then transferred to CCU for further management including therapeutic heparin infusion. The patient has no improvement in consciousness. CT head showed septum pellucidum hemorrhage and broke into bilateral ventricles, brain edema with herniation formation (Fig. 4E), given the poor prognosis, the patient was transitioned to comfort care after family discussion.

**Table 1:** The clinical manifestation of the three cases.⁎

Case	Sex	Age	Past Medical History	Risk Factors		Wells Score	Geneva Score	POCT				Point of care ultrasound		
				Tumor	Trauma			D Dimer (ng/ml)	CTnI (pg/ml)	BNP (pg/ml)	ECG	RV/LV	D shape sign	Visual thrombus
P1	Male	49	Hypertension	N	Y	4	4	2.53	23	1272	Sinus tachycardia, ischemic changes	>1	Y	N
P2	Male	65	Hypertension, atrial fibrillation	N	N	1	2	4.1	39	3980	Atrial filtration, ischemic changes	>1	Y	Y
P3	Male	57	None	Y	N	3	4	3.8	11	1022	Sinus tachycardia, premature ventricular contraction, ischemic changes	>1	Y	Y

Well Score:0–1 low risk; 2–6 moderate risk, >4 high risk.

Geneva Score:0–3: low risk; 4–10: intermediate risk; ≥ 11: high risk.

⁎ EKG after ROSC was achieved.

### 3.3 Case 3

**Fig. 5:**
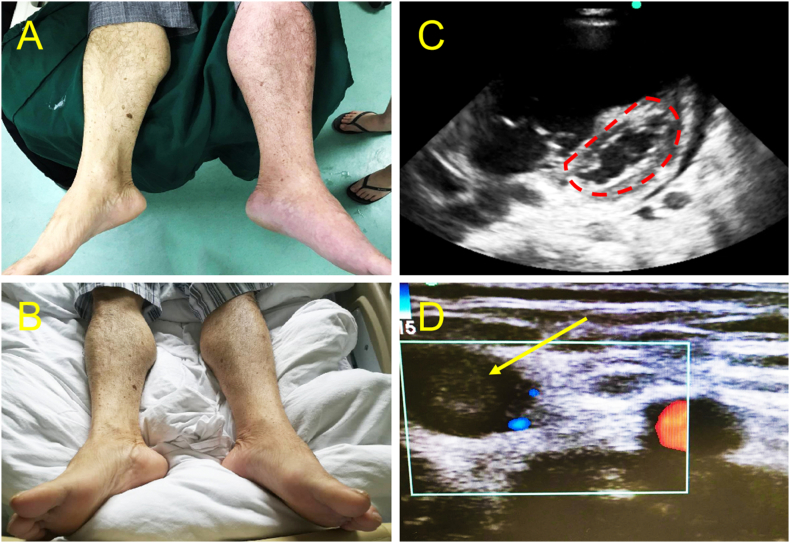
Lower extremity point-of-care ultrasound of patient 3. A: Cyanosis noted on left lower limb before thrombolytic therapy; B: improvement of discoloration on left lower limb after thrombolytic therapy, C: parasternal short axis shows D shape sign; D: visible thrombosis noted in left femoral veins.

**Fig. 6:**
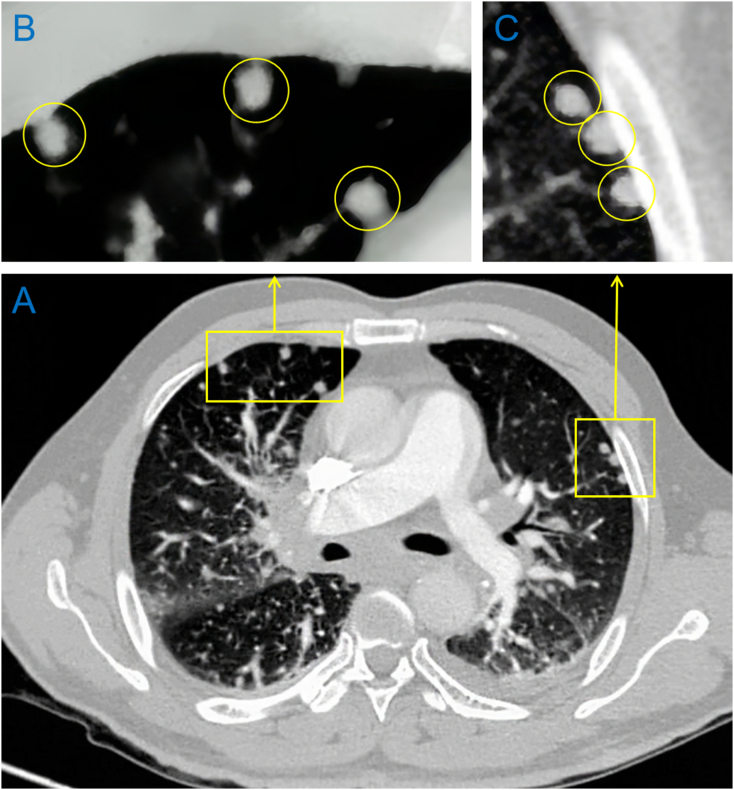
The CTA chest of patient 3 after thrombolytic therapy. A: multiple nodules in the lungs; B, C: zoom-in view of lung nodules.

A 57-year-old gentleman without significant past medical history, presented to the emergency department with acute right lower limb edema and pain and discoloration (Fig. 5A), which radiates from the shin to the thigh, with tingling. The symptoms progressively worsened. On arrival, D- D-dimer was high at 3.8 μg/ml (Table 1), CTnI and BNP were negative, patient was started on a therapeutic dose of enoxaparin, however, his symptoms continued to progress, he developed chest tightness and dyspnea, shock and altered mental status, BP 67/71 mmHg, SaO2 dropped to 63 %, the patient developed ventricular fibrillation, for which he was started on CPR and cardioversion (T + 0:00), ROSC was achieved, given the clinical picture, diagnosis of acute pulmonary thromboembolism was considered, point-of-care ultrasound was obtained during CPR which showed D shape sign (Fig. 5C) and left iliac venous thrombosis (Fig. 5D) further confirmed our diagnosis. The patient developed recurrent cardiac arrest afterward, he was given CPR and systemic thrombolytic therapy (T + 0:12). The patient achieved ROSCT (T + 0:26)， converted to sinus rhythm, BP improved, and became more alert, the discoloration improved gradually (Fig. 5B). CTA was obtained (T + 3:20) when the patient was stable, which shows multiple lung nodules and lymphadenopathy in the mediastinum but no significant filling defect (Fig. 6). He was admitted to CCU for further management. On day 2, there is still multiple thrombosis noted on lower limb veins according to venous duplex. The patient was transferred to the ward with significant improvement, he was later diagnosed with lung adenocarcinoma via lung biopsy.

This study has been reported in line with the PROCESS 2023 criteria [4,6].

## 4 Discussion

Cardiac arrest it's the most dangerous comorbidity of acute pulmonary thromboembolism and high mortality. Many patients with PTE were not diagnosed till autopsy given the duration of symptoms onset to death is very short [4,6]. Therefore, the key of management is to identify the diagnosis as soon as possible. For those patients with hemodynamically instability or even cardiac arrest, CTA usually could not be done in a timely manner, which challenges the diagnosis a lot.

This case series reported three cases with cardiac arrest, CTA could not be done immediately, point-of-care ultrasound had to be done during the CPR, which provided support for our diagnosis, The finding to diagnosis acute pulmonary thromboembolism was applicable even during the CPR [7].However, in the process of continuous cardiopulmonary resuscitation, transthoracic ultrasound has certain limitations. First of all, it should not affect the quality of CPR, and the duration of ultrasound examination should not exceed 10 s each time CPR is interrupted. The images obtained may also be fuzzy, requiring the examiner to replay them [9].It was point out in the guideline of European Society of Cardiology that point-of-care ultrasound could be used for the diagnosis if CTA is not obtainable. Compared to transthoracic ultrasonography, transesophageal ultrasonography was more advanced given its better solution without causing interruption of CPR [8]. However, given the cost and limitations of the exam, transthoracic sonography is still the most common way. Typical findings of acute pulmonary thromboembolism include enlargement of right ventricle, D shape sign, tricuspid valvular regurgitation, pulmonary hypertension. Given the exam was performed during CPR use the most accessible window was used, which showed the enlargement of right ventricle, RV/LV ratio, typical D sign as our major diagnosis tool in order to maintain the quality of CPR and minimize to interruption to <10 s. Although tricuspid valve regurgitation and pulmonary hypertension are more quantitative factors, they are time-consuming to obtain, which was not an ideal choice for the case of cardiac arrest. It was believed D shape during systolic phase was more meaningful for diagnosis, turning to replay function and play speed adjustment on ultrasound may be used. This is because during diastolic phase, pulmonic valve closed and tricuspid valve opened, the pressure of right ventricle is more related to the volume load itself, while the D shape during systolic phase is the opposite, which is more from the blocked pulmonary artery.

In terms of diagnosis, EKG and CTnI are the important tools to rule out acute myocardial infarction, among all the three cases, EKG did shows sinus tachycardia and myocardial ischemic change, which could be a finding of acute pulmonary thromboembolism but not specific one. The EKG is case 2 is obtained after ROSC achieved, CTnI, as the marker for cardiac injury, was not elevated significantly. However, there is a chance that cardiac arrest developed during the hyperacute phase of myocardial infarction, in which case CTnI but not be so high as expected, so myocardial infarction still cannot be entirely ruled out. D- Dimer is associated with acute pulmonary thromboembolism, but not specific, also seen in other cases including malignancy [9], like case 3. Moreover, other risk factors are limited to access given acuity of disease and the Well Score and Geneva score may also have their limitations when it comes to patients with cardiac arrest, in which case heart rate is not applicable. Other risk factors are limited due to the acuity of the disease and the patient's mental status. Therefore, the appropriate candidate for systemic thrombolytic therapy with diagnosis under POCUS are those with cardiac arrest or peri-arrest period, with acute right ventricular systolic pressure, which could be manifested with right ventricular dilation or diastolic collapse, with visible thrombosis in ventricles or deep veins of the lower extremities.

Given the uniqueness of the patient population, limited studies have been done to evaluate the outcome of pulmonary embolism-related cardiac arrest with or without thrombolytic therapy. One study showed the survival rate of pulmonary embolism-related cardiac arrest is not superior to non-pulmonary embolism cardiac arrest (68.3 % versus 64 %), even with thrombolytic therapy [10]. When it comes to the comparison between with and without thrombolytic therapy, one retrospective study failed to show a significant difference in the survival rates [11]. Another retrospective study showed thrombolytic group had a better survival rate after 24 h((19/36 (53 %) vs. 7/30 (23 %), *P* = 0.01) and survival to discharge (7/36 (19 %) vs. 2/30 (7 %))， although the latter was not statically significant (*P* = 0.15). The small sample sizes in these two studies may limit the power to detect significant findings. In comparison, a meta-analysis showed the superiority of mortality reduction in the thrombolytic group when it compared to the heparin-only group for massive and submassive PE [12]. The same study also showed a better outcome in some benefits in long-term prognostic factors like pulmonary hypertension and composite outcome. It is challenging to reverse cardiac arrest, merely with CPR, when it is secondary to acute pulmonary thromboembolism. Reperfusion with CPR is the key to management of pulmonary embolism-related cardiac arrest on guidelines [13]. So far, reperfusion for acute pulmonary thromboembolism includes systemic thrombolysis [14], interventional radiology-guided thrombolysis, IR guided or surgical thrombectomy, with or without the support of ECMO [15]. Although other alternative may have a better safety profile for non-cardiac-arrest patients [16], this may not be applied to those who already had cardiac arrest. One study showed that for those patients who received surgical thrombectomy after cardiac arrest, the mortality is 44.4 % [17]. In terms of time taken for thrombolysis and accessibility of treatment, systemic thrombolytics show their superiority compared to others. Among the three cases presented, the patient received cardiac arrest right at the time of the identification of cardiac arrest, the cardiac arrest to thrombolytic windows are respectively 6 min, 13 min, 12 min, while the time taken for ROSC is 19 min, 33 min and 226 min. It is reported that for acute pulmonary thromboembolism, it takes almost 20–30 min from thrombolysis to reperfusion [18]. Therefore, it is reasonable to consider prolonging CPR for cardiac arrest caused by pulmonary embolism to achieve reperfusion. There are scarce studies evaluating the long-term outcome if patient population is limited to patients with cardiac arrest. One small-sized randomized control study failed to show difference in neurological outcome at 30 days [19]. But maybe it reasonable to consider cautiously applying the conclusion of the studies of massive and submassive PE, which suggested a favorable long term outcome, to patients with pulmonary embolism related cardiac arrest.

One of the major concerns of systemic thrombolytics during CPR, is the risk of bleeding, especially intracranial bleeding. In terms of medications, rtPA was chosen, for the total dosage are all 100 mg, but in terms of the initial dose, we used 10 mg intravenous bolus as for case 1 and case 3, while for case 2 50 mg was given since the patient had cardiac arrest before arrival. This was supported by previous studies, which suggested enhanced survival and reduction in pulmonary artery pressure [14]. The patient in case 1 developed diffused gastrointestinal bleeding, which could be attributed to his use of high-dose nonsteroid anti-inflammatory drugs for his fracture, The patient, in case 2, unfortunately, developed intracranial hemorrhage; his prognosis is poor given his age, duration for CPR and intracranial hemorrhage. This may indicate the importance of individualizing the dose of tPA for patients. There are studies indicating half dose (50 mg) of rtPA may show similar efficacy but lower risk of bleeding for high-risk pulmonary thromboembolism [20,21], however, this may not apply to patients who have already developed cardiac arrest, whose circulation is presumably poor.

## 5 Conclusion

It is crucial to identify cardiac arrest caused by acute pulmonary thromboembolism, in the circumstances where CTA could not be done, point-of-care ultrasound is a reliable tool to be used. Systemic thrombolytic therapy with CPR is an effective means of treatment, which may increase the successful rate for ROSC significantly.

## Consent

Written informed consent was obtained from the patient for publication of this case report and accompanying images. A copy of the written consent is available for review by the Editor-in-Chief of this journal on request.

## Ethical approval

The ethical committee approval was not required given the article type (case report).

## Guarantor

Lifeng Xiao.

## Research registration number

Not applicable.

## Funding

This research did not receive any specific grant from funding agencies in the public, commercial, or not-for-profit sectors.

## Author contribution

**Lifeng Xiao** is the Principal Investigator of this study and is responsible for conceptualization and methodology and performed all the surgeries and follow-up of the patient as well as contributed to writing of this manuscript.

**Qishuo Zhang, Xiaomin Ou and Wanshan Liu** drafted the work and contributed to data curation, conceptualization, and writing of the manuscript. RI interpreted and analyzed the pathological figures.

All authors have read and approved the final manuscript.

## Declaration of competing interest

The authors state that they have no conflicts of interest for this report.
